# The effect of intravenous dexamethasone in the treatment of septic arthritis

**DOI:** 10.12669/pjms.305.5217

**Published:** 2014

**Authors:** Hamidreza Arti, Ali Mousapour, Sayed Mohamad Alavi

**Affiliations:** 1 Dr. Hamidreza Arti, Associate Professor, Orthopaedic Surgery Department, Imam Khomeini Trauma Research Center, Ahvaz Jundishapour University of Medical Sciences, Ahvaz, Iran.; 2 Dr. Ali Mousapour, Resident of Orthopaedic Surgery Department, Ahvaz Jundishapour University of Medical Sciences, Ahvaz, Iran.; 3 Dr. Sayed Mohamad Alavi, Professor of Infection and Tropical Department Disease, Ahvaz Jundishapour University of Medical Sciences, Ahvaz, Iran.

**Keywords:** Septic Arthritis, Dexamethasone, Inflammation

## Abstract

***Background and Objective:*** Septic arthritis is a joint infection that causes metabolic and physiological disorders and if not diagnosed and treated on time can cause severe damage and disabilities. In this study, the effect of intravenous dexamethasone on septic arthritis, and the recovery process of the disease have been evaluated.

***Methods: ***In a non-randomized double blind clinical trial 60 patients assigned in two groups of 30 patients each were evaluated. After diagnosis of septic arthritis, group one received a dose of 0.15mg / kg / QD of dexamethasone and group two received the same amount of normal saline for four days. Then the patients were evaluated for treatment duration, inflammation and redness relief, joint movement and ESR and CRP levels.

***Results: ***After data collection they were compared with the X2 test, t-test, ANOVA and Mann-Whitney statistical test and were analyzed using SPSS18 software. Treatment duration was 3.27 ± 1.04 days, joint movement was 50.67 ± 9.7 degrees, inflammation and redness relief was seen after 4.1 ± 0.32 days, a decrease of 19.3 ± 2.89 in ESR levels were seen and reduction in CRP levels showed a statistically significant difference (P <0.05).

***Conclusion:*** In patients with septic arthritis in addition to routine antibiotic therapy, receiving intravenous dexamethasone can reduce the clinical symptoms of the disease symptoms and also accelerate recovery and return to daily activities.

## INTRODUCTION

Septic arthritis or infection of the joints is the invasion of a joint by a pathogen or disease-causing micro-organism that causes metabolic and physiological disorders of the joint. If not diagnosed and treated in time it can lead to severe and debilitating injuries and cause about 25% disability after development, that can include bone deformities, limitation of motion, instability and dislocation. Any of these effects can develop months or years after the disease occurs.^1^^-^^5^

Staphylococcus aureus is the most common pathogen in this disease and is considered the most important inflammation causing organism. Even when these microorganisms have been effectively eradicated with antibiotics, the inflammatory process in the joint is continued, leading to delayed recovery and other joint injuries.^6^

In septic arthritis, high concentrations of inflammatory cytokines in the joint can be seen. Several studies have shown that the increasing rate of two cytokines in the serum including IL-1and TNF-α have been associated with a significantly increased severity of septic arthritis in children.^1^^,^^6^^-^^8^

Dexamethasone is an adrenocortical steroid known as an anti-inflammatory drug. The inhibitory effects of dexamethasone on prostaglandins and enzyme production improve or prevent inflammation.^3^^-^^5^

According to studies performed on human and others on animals, intravenous administration of dexamethasone along with antibiotics in patients with septic arthritis can have an anti-inflammatory effect and can reduce the rate of injuries and complications, and these severity of the disease.^3^^,^^9^

Due to the lack of published studies worldwide and the importance of septic arthritis as a disabling disease and its prevalence, and also, the role of medications and effective surgical procedures in treating and preventing disease complications we conducted a study to evaluate the effect of dexamethasone administration on the treatment of septic arthritis in children.

## METHODS

During a double-blind randomized clinical trial, patients admitted to hospitals from 2010 to 2012 were studied. All parent's patients signed an informed consent form after approval of ethical committee and registered in IRCT (NO: IRCT 2013112011014N1). Inclusion criteria included 1) confirmed septic arthritis, 2) age above six months, 3) signed informed consent by parents for participating in the study. Exclusion criteria included 1) immunodeficiency or leukemia, 2) concurrent septic disease, 3) use of antibiotics before culture, 4) use of corticosteroids, 5) systemic inflammatory diseases such as rheumatoid arthritis or systemic lupus erythermatosus. In this study, patients were divided into two groups of 30 patients and a sample of joint fluid and blood samples for each patient was sent for culture. After hospitalization, patients received routine intravenous antibiotics for three weeks and surgery was scheduled for aspiration or drainage of affected joints. Dexamethasone 0.15 mg / kg / QD was administered in group one over four days and the over the same period and same volume of normal saline was administered group two. Patients in both groups were compared on the basis of clinical and laboratory findings. Clinical findings included inflammation and redness of the joint, the amount of motion of the affected joint and the duration of therapy and laboratory findings included ESR and CRP levels. Patients were followed up after treatment. Data was analyzed with the X2 test, t-test, ANOVA and Mann-Whitney statistical test and software Spss. 18 P <0.05 was considered significant.

## RESULTS

In group one, the mean age was 8.06 ± 0.5 years, 73% participants were male and 27% were female and the mean age in group two was 8± 0.6 years old, 70% participants were male and 30% were female. Mean age was not significantly different between the two groups (P> 0.5). In terms of clinical findings of septic arthritis after administration of dexamethasone, the number of days of inflammation and redness of the patients in group one was 2.43 ± 0.15days and in group two was 6.53 ± 0.27days,the difference between the two groups was significant (P = 0.0014). The number of days of hospitalization in group one was 8.9 ± 0.88 days and group two was 12.17 ± 0.54 days with significant difference between the two groups (P = 0.005). The amount of motion of the affected joint in group one was 91.2 ± 2.28 degrees and in group two was 40.53 ± 1.44 degrees, the difference between the two groups was significant (P = 0.008). Laboratory findings included a reduction of ESR levels, 36.03±2.77in group one and 16.73 ±0.83 in group two with a significant difference between the two groups (P = 0.0001). Reduction of CRP levels with use of X2 test, t-test, ANOVA and Mann-Whitney statistical test was considered statistically significant (P <0.05).

## DISCUSSION

The results of this study indicate that the addition of dexamethasone to antibiotic therapy regimens in patients with septic arthritis can lead to accelerated recovery. Group one compared with group two had less duration of local and systemic symptoms, and acute phase reactants were suppressed at shorter periods. Treatment with intravenous dexamethasone can decrease the inflammation and redness of joints involved, duration of hospitalization, ESR and CRP levels. The intervention also showed improvement in joint function and range of motion after the treatment course. The weaknesses of this study were lack of measurement of inflammatory cytokines including TNF-α, IL-β, chondroitin sulfate and metalloproteinases in synovial fluid. Measurements have shown that the metabolism of these factors effect bacterial infection of the joint cartilage and pathogenic processes.^10^ Other weaknesses included not using the CRP factor as a quantitative variable, a beneficial effect of dexamethasone on the treatment period of septic arthritis due to the limited number of patients in this study could not be demonstrated and the prognosis of the different strains is equal. Also the lack of pursuing clinical and laboratory symptoms during successive periods after treatment resulted in the impossibility to evaluate the long term effect of dexamethasone on long-term prognosis of septic arthritis.

**Table-I:** Comparing the effect of Dexamethasone in treatment results of septic arthritis as Means±SD in two groups

***Group***	***Days of Hospitalization***	***Days of Inflammation***	***Joint Motion***	***ESR***
Case	8.9±0.88	2.43±0.15	91.2±2.28	36.03±2.77
Control	12.17±0.54	6.53±0.27	40.53±1.44	16.73±0.83

As other researchers have also proven, measurement of CRP levels of serum is a useful prognostic index for assessing the response to treatment, and the level of this factor decreases faster in patients undergoing dexamethasone therapy. Faster decrease of CRP in the case group expresses the fact that IL-6 cytokine, which is responsible for CRP elevation, acts along with IL-1, TNF-alpha and beta, because this cytokines also decrease with the use of dexamethasone.^10^ Before 2003, most studies were performed in a laboratory setting on animals, for example in a study by Sakiniene et al. in 1996, the effect of corticosteroids in addition to antibiotic therapy on the severity of septic arthritis caused by staphylococcus aureus in mice was studied and showed that this protocol decreased the severity and mortality and morbidity due to the disease.^9^^,^^10^ Additionally, two other studies showed the protective effect of corticosteroids on cartilage in septic arthritis in rabbits, provided the timely use of systemic antibiotics.^10^^,^^11^ Of studies performed on humans, the study of 123 children with septic arthritis in 2003 by Odio et al. can be noted in which early use of dexamethasone leaded to accelerated healing periods and a decrease in bacterial septic arthritis dysfunction at the end of the treatment period was 26% that was close to other studies(23%).^10^^,^^11^ Furthermore, in 2011 Mr. L. Harel noted that in a double blind randomized study on 49 children with septic arthritis, the number of days of inflammation was 2.43±0.15 days and the number of days of hospitalization was 9.8±0.88 days in group one that these parameters in comparison to the group evaluated in this study were 5.5±6.6 and 9.91±4.84 days.^11^

Intravenous administration of dexamethasone with antibiotics in patients with septic arthritis leads to acceleration in healing time, decrease in duration of treatment and a better prognosis.
